# Molecular detection of lumpy skin disease virus in naturally infected cattle and buffaloes: unveiling the role of tick vectors in disease spread

**DOI:** 10.1007/s11259-024-10541-7

**Published:** 2024-10-08

**Authors:** Gamil S. G. Zeedan, Abeer M. Abdalhamed, Ahmad M. Allam, Sobhy Abdel-Shafy

**Affiliations:** Department of Parasitology and Animal Diseases, National Research Centre, 33 Bohouth Street, Dokki, Giza, 12622 Egypt

**Keywords:** Lumpy skin disease virus, *Rhipicephalus annulatus*, Ticks, Poxviruses, Multiplex PCR, Rt-qPCR, Cattle, Buffaloes

## Abstract

Lumpy skin disease (LSD) is a viral disease that affects cattle and buffaloes in Egypt, causing considerable economic losses in the animal sector. This study aimed to investigate the recent outbreak of LSDV in cattle and buffaloes and evaluate the potential role of the hard tick *Rhipicephalus annulatus* in their transmission through isolation and molecular characterization by multiplex PCR (mPCR) and real-time quantitative PCR (rt-qPCR) assays. A total of 50 skin biopsies (cattle *n* = 30, buffaloes *n* = 20), 110 nasal swabs (cattle *n* = 76, buffaloes *n* = 44), and 129 blood samples (cattle *n* = 84, buffaloes *n* = 45) were collected. In addition, 145 hard ticks of different stages were collected from cattle and buffaloes of different breeds and ages in different governorates in Egypt from November 2021 to June 2022. Multiplex PCR and real-time quantitative PCR (rt-qPCR) assays based on SYBR Green and targets (P32, VP32, G protein, and viral fusion protein) were used. We identified positive results in 17 out of 30 cattle skin biopsies (56.6%), 1 out of 7 buffalo skin scabs (14.3%), and 5 out of 45 buffalo blood samples (11.11%) using mPCR and RT-qPCR methods. We successfully isolated LSDV from hard ticks and cattle infested with ticks and exhibited characteristic signs of LSD on the chorioallantois membrane (CAM) of specific pathogen-free embryonated chicken eggs (SPF-ECE). The isolates were confirmed by multiplex PCR and RT-qPCR. The cyclic threshold (Ct) with correlation-slandered curve values of rt-qPCR ranging from 10.2 to 36.5 showed the amount of LSDV-DNA in different samples. The study's findings demonstrated the widespread circulation of LSDV in both cattle and buffaloes in Egypt and provided strong evidence that hard ticks *R. annulatus* play a role in the transmission of LSDV in susceptible animals.

**Supplementary information:**

The online version contains supplementary material available at 10.1007/s11259-024-10541-7.

## Introduction

Lumpy skin disease (LSD) is a contagious viral skin disease that affects cattle. It belongs to the genus *Capripoxvirus* (CaPV), subfamily *Chordopoxvirinae,* within the *Poxviridae* family (Buller et al. 2005; WOAH 2023; Mashamba 2020; Gehring et al. 2023). LSDV is closely genetically and morphologically related to sheep and goat poxviruses (Zeedan et al. 2014, 2019). It causes an acute disease in cattle, characterized by elevated body temperature, skin nodules, skin swelling, weight loss, and lymph node enlargement (Gammada et al. 2022). LSD inflicts substantial economic losses due to skin damage, decreased milk production, infertility, and restrictions on international animal trade (Zeedan et al. 2020; Jena et al. 2022). The spread of the LSD virus over long distances is associated with the movement of both visibly and invisibly infected cattle through different means of transportation, such as road, rail, or foot, directly toward markets or seasonal grazing areas. The periodic nature of outbreaks suggests a probable correlation between local virus transmission and the abundance of insect vectors (El-Ansary et al. 2022). Mechanical transmission of LSDV by arthropod vectors, including mosquitoes such as *Anopheles stephensi, Aedes aegypti, Culex quinquefasciatus and Culicoides nubeculosus* (El-Ansary et al. 2022). Although ticks are recognized as vectors of pathogens and primary carriers of disease agents in animals, the role of ticks as biological vectors for LSDV has not been extensively studied (Lubinga et al. 2013; Aleksandr et al. 2020). Recently, molecular detection has indicated the transmission of LSDV by *Rhipicephalus annulatus* (*R. annulatus*) ticks in both the transstadial and transovarial routes (Lubinga et al. 2013). Additionally, there has been documented that the LSDV is transmitted by mechanical transmission by male ticks of *Amblyomma hebraeum* (*A. hebraeum* ) have shown transstadial transmission after molting from nymphs previously fed on experimentally infected cattle (Lubinga et al. 2015). Lumpy skin disease has expanded its presence to the Middle East, initiating outbreaks in Egypt's Ismailia governorate during the summer of 1988 (Elhaig et al. 2017; Abdallah et al. 2018; Selim et al. 2021). Subsequent outbreaks occurred in Saudi Arabia (1990), Kuwait (1991), Lebanon (1993), the United Arab Emirates (2000), and Oman (2010) (Jena et al. 2022). The prevalence of LSDV persisted in the Middle East until the summer of 2022 (Azeem et al. 2022), with the summer being a common period for LSD outbreaks in these regions (Rouby et al. 2017; Fawzi et al. 2022; Hamza et al. 2023).

Diagnosis of LSDV involves several methods, including viral isolation in embryonated chicken eggs (ECE), tissue culture, and agent identification techniques such as polymerase chain reaction (PCR)-based assays. The serological diagnosis of LSD involves indirect enzyme-linked immunosorbent assays (I-ELISA), indirect fluorescent antibody techniques (IFAT), and Western immunoblotting (WB). However, a drawback of serological tests is their inability to distinguish between infected and vaccinated animals or antibodies produced by LSDV infection from those of other poxviruses (Zeedan et al. 2019; Yimer 2021). Molecular diagnosis of LSDV has been developed using different PCR methods, as real-time PCR has a highly effective method, offering speed, closed systems, and no need for post-PCR electrophoresis, reducing contamination risks, being specific and highly sensitive, and facilitating the detection and analysis of mutations, including single nucleotide polymorphisms (Vidanović et al. 2016;  Dubey et al. 2023). Genotyping using rt-qPCR can be achieved without multiplexing by observing the melting point temperature (Tm) between the probe and its target, which varies for each strain. The melting peaks of the post fluorescence melting curve analysis help differentiate among vaccinal strains of LSDV, virulent LSDV isolates, sheep pox (SPP), and goat poxviruses (Yimer 2021). LSDV reemerged in Egypt despite attempts to control the disease through vaccination with the Romanian sheep pox virus vaccine (Rouby et al. 2017; Ahmed et al. 2021; Fawzi et al. 2022; Hamza et al. 2023). A Neethling strain isolated in South Africa was successfully attenuated and used. The vaccine was shown to be harmless and immunogenic, though some local reactions have been observed in some animals. The vaccine was used in six Balkan countries during 2016–17 (Bulgaria, Greece, Serbia, Montenegro, the former Yugoslav Republic of Macedonia, and Albania), and the average ratio of its effectiveness was 79.8% (range = 62.5–97%) (Morgenstern and Klement 2020). This led to the development of a homologous vaccine based on the *Neethling strain* (MEVAC®), evaluated for safety and effectiveness during emergency periods in Egypt and Vietnam from 2020 to 2021 (Bazid et al. 2023). This study aimed to investigate the recent outbreak of LSDV in cattle and buffaloes and evaluate the potential role of hard ticks (*R. boophilus* *annulatus)* in their transmission through isolation and molecular characterization by multiplex PCR and rt-qPCR assays.

## Materials and methods

### Sample collection

A total of 50 skin biopsies (cattle *n* = 30, buffaloes *n* = 20), 110 nasal swabs (cattle *n* = 76, buffaloes *n* = 44), and 129 blood samples (cattle *n* = 84, buffaloes *n* = 45) Furthermore, 145 hard ticks of different stages were collected from cattle and buffaloes of different breeds and ages in different governorates in Egypt from November 2021 to June 2022 in different Egyptian governorates (Beni-Suef, El Fayoum, Giza, Monefia, Sharqiya, Marsa Matrouh, and Gharbiya). Cattle suffer from fever, with the appearance of firm nodules throughout the body of the animals. Furthermore, buffalo samples were collected aseptically from the skin, as shown in Table 1. Each of these samples was kept in a sterile screw-capped tube and transported on an ice box to the laboratory for viral isolation, multiplex PCR, and real-time PCR.

**Table 1:** Collection of Samples from Cattle and Buffaloes During Lumpy Skin Disease Virus (LSDV) Outbreak in Various Governorates of Egypt. All suspected cases were clinically examined, and the hard tick *R. annulatus (formerly Boophilus*) was collected from the cattle and buffaloes with suspected LSDV infection. Live ticks were morphologically identified using taxonomic keys at National Research Centre, Dokki, Egypt

*Location Governorates*	*Species*	*No*	Skin biopsy	*Nasal* *Swabs*	*Ticks* samples *	*Blood samples*	
						Sera	W. blood
*Beni-suef*	cattle	20	6	11	25 / 5 G	20	20
	buffaloes	5	3	5	15 / 3 G	5	5
*El Fayoum*	cattle	15	5	15	17/3 G	15	15
	buffaloes	10	3	10	6 / 1G	10	10
*Giza*	cattle	13	5	6	18 / 3G	13	13
	buffaloes	10	4	10	4 / 1G	10	10
*Monefia*	cattle	3	2	3	13 / 3 G	3	3
	buffaloes	2	1	2	6 / 1 G	2	2
*Sharqiya*	cattle	16	7	16	13 / 3G	16	16
	buffaloes	7	3	7	3 / 1 G	7	7
*Marsa Matrouh*	cattle	3	2	2	6 / 1 G	3	3
	buffaloes	2	1	1	6 / 1 G	2	2
*Gharbiya*	cattle	14	3	13	9 / 2G	14	14
	buffaloes	9	5	9	4 / 1 G	9	9
*Total*		129	50	110	145 /29 G	129	129

Ticks’ samples*: divided into group each of them ranged from 3 to 5 ticks according to location and LSD signs

W. blood: Uncoagulated blood with EDTA No: number

### Tick samples

A total of 145 ticks from different stages were collected from different suspected cattle and buffaloes with or without LSD signs. The tick samples were divided into 29 pools (each containing 3–6 ticks), washed twice with sterile water to remove contamination, and divided in half. One of them was washed once with 70% ethanol, cut into small pieces, and ground with buffered phosphate saline (PBS) using a sterile mortar and pestle. For homogenization of egg samples, eggs were sonicated using an ultrasonic processor with a 2 mm microtip probe. The optimum sonication was 4 cycles of 3 sec. bursts and a 5 sec. interval between bursts. The homogenate was centrifuged at 600 g for 5 min, and the supernatant was collected and stored at -80 °C to be used for PCR and virus isolation. They were morphologically identified according to Walker et al. (2003) and Estrada-Peña et al. (2006) using taxonomic keys and light microscopy in the laboratory of the Department of Parasitology and Animal Diseases, National Research Centre, Cairo, Egypt.

## Experiment design for LSDV transmitted to tick eggs and larvae

**Scheme 1:**
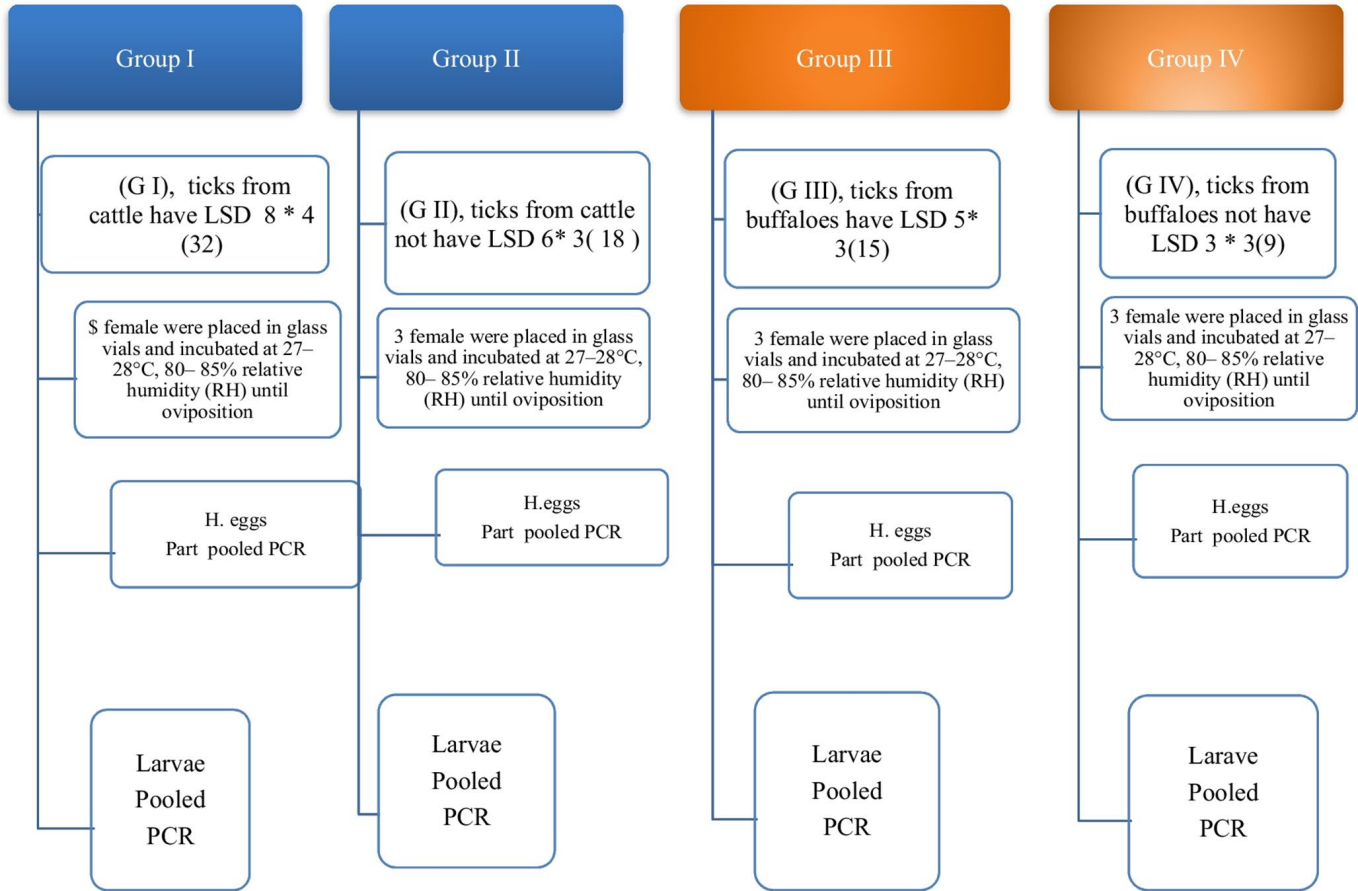
Experiment design for proved biological LSDV transmitted from ticks to their eggs and larvae. This experiment investigates the potential transmission of Lumpy Skin Disease Virus (LSDV) through ticks by infecting ticks with the virus and analyzing their eggs and larvae for its presence. 74 engorged ticks, collected from both cattle and buffaloes with and without LSD, were divided into four groups based on origin and infection status. Ticks were incubated, allowed to lay eggs, and sampled for viral analysis. Egg pools and individual larvae were then further analyzed for LSDV presence. By examining eggs and larvae, this study aims to provide insights into the possibility of transovarial and early-stage transmission of LSDV within tick populations, with potential implications for disease control strategies in both cattle and buffalo populations

A total of 74 fully engorged live female ticks collected from cattle and buffalos with signs of LSD or without signs (asymptotic animals) were washed with normal saline pH 7.2, then divided into groups as shown in Scheme 1. Each group contained 3–5 ticks, which were then placed in flacon vials as shown in Scheme 1 and Table 1. The collected ticks were distributed on the day of collection into four groups, each subjected to a different group as follows: Group I (G1): ticks from cattle have LSD; group II (G2): ticks from cattle do not have LSD; group III (G3): ticks from buffaloes have LSD; and group IV (G4): ticks from buffaloes do not have any LSD. Incubated at 27 to 28 °C with 75 to 80% relative humidity (RH) until oviposition (Castro-Janer et al. 2009). Samples were taken from each egg mass and then pooled and subjected to DNA extraction and virus isolation. The other egg masses were individually stored at 27–28 °C with 75–80% relative humidity (RH) until the larvae hatched.

### Viral DNA extraction

Thirty skin nodules, blood, nasal swabs, and 29 pooled tick samples were extracted using the QIAamp® DNA Mini Kit for DNA extraction from blood, NS, and skin biopsies (QIAGEN GmbH, Germany) following the manufacturer’s instructions. System (Bio-Rad) and compared with a positive control (SPPV vaccine and LSDV) and a 100-bp DNA marker.

### Real-time qPCR based on SYBR green dye

A plate-multiplex quantitative rt-qPCR assay of SYBR Green (RT2 SYBR® Green qPCR Master mixes, QIAGEN) was performed, targeting P32, VP32, G protein, and viral fusion protein genes, in 20 μL containing 0.5 μL of each primer, a master mix of Syber Green, and 2 μL of DNA template. initial temperature of 95 °C for 30 s, followed by 40 cycles of 95 °C for 5 s, 60 °C for 30 s, and 72 °C for 30 s. The sample was considered positive if the cycle threshold level was greater than the Ct value. The qPCR conditions involved 1 cycle of pre-denaturation (95 °C for 5 minutes), 40 cycles of denaturation (95 °C for 20 s), annealing and extension with detection (58 °C for 1 minute), and a melt cycle (65–90 °C). The qPCR data analysis was carried out in QuantStudio 5 Real-Time PCR System software by calculation of the threshold cycle number (Ct value) for the positive amplification and negative control samples.

### TaqMan real-time PCR

The mixing step was performed using a commercial qPCR kit (LSDV dtec-qPCR kits; GPS™, Alicante, Spain) compared to rt-qPCR-SYBR Green, as shown in Table 2. The contents of the mix kit were rehydrated and used according to the manufacturer. The reaction was prepared in sterile real time PCR tube strips with a final volume of 20 μl prepared and thoroughly mixed by pipetting (4μl mix stable qPCR 5x, 1μl specific primer/probe, 10 μl DNase/RNase free water, and 5μl template (sample, positive or negative). The mix was then applied to the Quik Sudio 5™ real-time PCR system with the following cycling conditions according to the manufacturer: 1 cycle at 95 °C for 15 min for activation, followed by 40 cycles at 95 °C for 15 s for denaturation, and 60 °C for 1 min for hybridization, extension, and data collection. The cutoff was determined by the manufacturer at the value of the 35 cyclic thresholds (Ct). Positive control was among the contents of the commercial kit.

**Table 2:** Primers sequences for P32 gene, VP32 gene GPCR gene, and VF gene viral fusion protein, includes amplicon sizes

No	*Tagert genes*	*Primers sequences*	TM	*bp*	*Ref*
1	*P32 gene in lumpy skin disease* *virus*	*CGCGAAATTTCAGATGTAGTTCCA* *TGAGCCATCCATTTTCCAACTC*	*55 C*	752 bp	Ireland and Binepal (1998)
2	*Viral attachment protein gene, VP32*	*TTTCCTGATTTTTCTTACTAT* *AAATTATATA C GTAAATAAC*	*50 C*	192 bp	Rouby et al. (2017)
3	*G-Protein coupled chemokine receptor (GPCR) gene*	*AGT ACA GTT AGT AGC GCA ACC* *GGG TGA ACT ACA GCT AGG TAT C*	*55 C*	554 bp	Hussein et al. 2017) El-Tholoth and El-Kenawy (2016)
4	*Viral fusion protein* (*VF*) *gene*	*TGTTGTACTTCGTCCTGTTTGAA* *CGACGATGATGAAACCAATG*	*50 C*	412 bp	Ireland and Binepal (1998)
5	P32 gene with probe	AAA ACG GTA TAT GGA ATA GAG TTG GAA AAA TGA AAC CAA TGG ATG GGA TA’ Probe FAM-TGG CTC ATA GAT TTC CT-3'		Real time - qPCR	

### Multiplex PCR

The PCR tube was 25 μL according to Diab et al. (2021), 2 μL of extracted DNA, 12.5 μL of master mix, and 1 μL of (20 pmol) of each forward and reverse primer gene (P32, VP32, G protein, and viral fusion protein), as shown in Table 2, and then completed up to a final volume with nuclease-free water. The PCR procedure consisted of an initial denaturation step at 95 °C for 10 minutes, followed by 40 cycles comprising denaturation at 95 °C for 55 seconds, annealing at 55 °C±5 °C for 55 seconds, and extension at 72 °C for 1.5 minutes. A final extension step was performed at 72 °C for 10 minutes. Visualization of the amplified PCR products was performed using a 1.5% agarose gel electrophoresis and a gel documentation system.

### Viral isolation

#### Viral isolation from skin biopsies

Skin biopsy specimens were minced using sterile scissors and forceps and then ground in a mortar with sand and an equal volume of sterile phosphate buffered saline (PBS), then subjected to virus isolation with an antibiotic mixture of streptomycin (1 mg/mL), penicillin (1000 IU/mL) and mycostatin (100 IU/mL) up to a final concentration of 10%. (W/V). The suspension was freeze-thawed three times, clarified by centrifugation at 1000 xg for 10 min, and then stored at -40 °C until use as described by Awad et al. (2010) and Abebe (2018).

#### Viral isolation from tick samples (adults, nymphs, and hatched larvae)

Tick samples were prepared according to Lubinga et al. (2013): washed with sterile distilled water, rinsed once with 70% ethanol, cut into small pieces, and ground with sterile PBS under aseptic conditions. Pooled tick samples were used to represent ticks from different areas, with each sample containing ticks. Each tissue homogenate was centrifuged at 6000 rpm for 10 minutes, subjected to DNA extraction, and examined by multiplex PCR. Positive samples were inoculated separately into the CAM of a 9–11-day-old ECE. All inoculated ECEs were incubated at 37 °C and 70% humidity. The incubated ECEs were examined daily for five days. Embryos that died during the first day after inoculation (PI) were discarded. CAMs were harvested and examined for characteristic pock lesions. CAMs that did not show pock lesions were subjected to one- or two-blind passages. The presence of LSDV was further confirmed by PCR and rt-qPCR.

#### References to virus strains

The reference LSDV strain (*Neethling strain*) is a freeze-dried, live attenuated LSD viral vaccine prepared from the Neethling strain of the virus in VERO cell culture, and the sheep pox vaccine against the Romanian strain (10 ^3.5^ TCID 50/dose) was purchased from the Veterinary Serum and Vaccine Research Institute, Abasia, Cairo, Egypt. Positive LSDV antiserum was kindly provided by the Animal Health Research Institute, Dokki, Giza, Egypt.

#### Endpoint enzyme-linked immunosorbent assay (EP-ELISA)

LSDV-antibodies were detected in blood samples of cattle and buffaloes using ELISA, according to Zeedan et al. (2019), with some modifications. The reference LSDV strain was propagated in the CAM of SPF-ECE. According to the method previously described, the supernatant of LSDV-infected CAM from SPF-ECE was centrifuged at 3000 rpm for 20 min. Then the supernatant was discarded, and the pellet was resuspended in phosphate-buffered saline (PBS) at pH 7.2 and lysed using a sonicator three times for 15 s each with a 1-minute rest (0.5 cycles, 80% amplitude). The suspension was centrifuged at 6500 rpm for 15 min at 4 °C to remove cell debris. The suspension was pelleted by centrifugation (Beckman Coulter) at 16,000 rpm in a SW-41 rotor at 4 °C for 60 min. After discarding the supernatant, the pellet was reconstituted in PBS (pH 7.2). Soluble LSDV proteins were subjected to a checkerboard titration against positive LSDV antiserum, kindly provided by the Animal Health Research Institute in Dokki, Giza, Egypt. The Spearman-Karber method was employed to determine the optimal concentration for coating the ELISA plate. A 96-well ELISA plate was coated with partial purified LSDV antigen diluted 1:100 in 0.05 M carbonate buffers, pH 9.6, then incubated overnight at 4 °C. The plate was washed three successive times with washing buffer (PBS, pH 7.2, containing 0.05% Tween-20) by the ELISA-Microplate Washer (Bio-Rad Microplate Washer). Added 100 μL of blocking solution containing 1% bovine serum album (BSA), then incubated for 1.5 hours at 37 °C. Then it was washed three times with a washing buffer. 100 μL of diluted tested serum (cattle and buffalo's serum) samples were diluted 1:50 in PBS, pH 7.2, containing 0.05% Tween-20. Control positive and negative serums were added, then incubated for one hour at 37 °C. Then it was washed three times with a washing buffer. 100 μL of anti-bovine horseradish conjugated horseradish peroxidase diluted at 1:3000 in PBS, pH 7.2, containing 0.05% Tween-20 according to the manufacturer’s instructions. The plates were then incubated for one hour at 37 °C. After incubation, the plates were washed three successive times in PBS, pH 7.2, containing 0.05% Tween-20, by the ELISA-Microplate Washer. 100 μL of orthophenylenediamine (OPD) substrate solution was added and incubated for 10–15 minutes in a dark place until a reaction was developed, then stopped by adding 50 μL of stopping buffer (2 M H2SO4). The ELISA plate was read at a wavelength of 492 nm using an ELISA reader (Bioteck ELX 808 IU Absorbance Microplate Reader, USA). The cutoff value used was higher than the mean optical density values of the control negative sera.

### Statistical analysis

Molecular assay analysis was performed using percentage and Fisher’s exact methods at a 95% confidence interval at *P* ≤0.05 using SPSS, version 16 (Chicago, Illinois, USA).

## Results

### Clinical signs of LSD in infected cattle

**Fig. 1:**
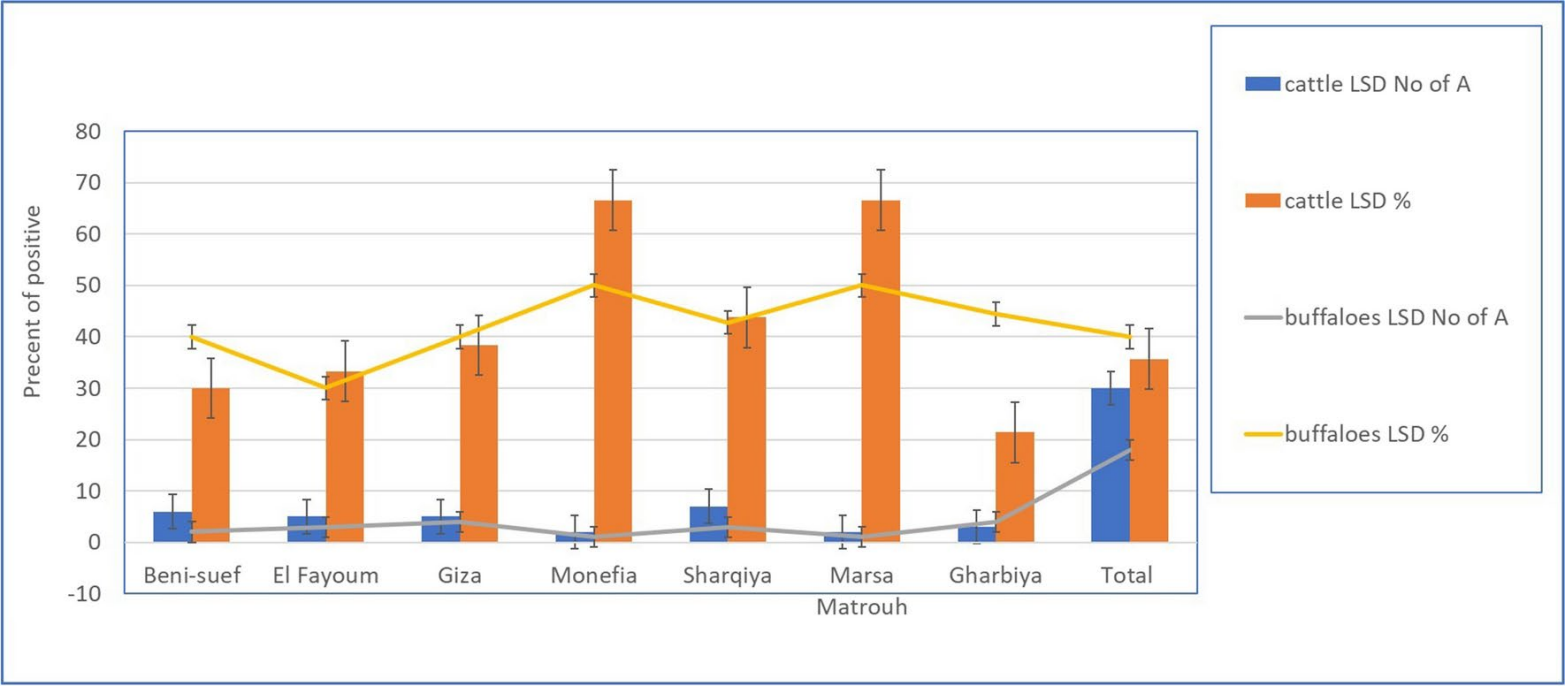
Clinical signs of LSD in examined animals in different Governorates

**Fig. 2:**
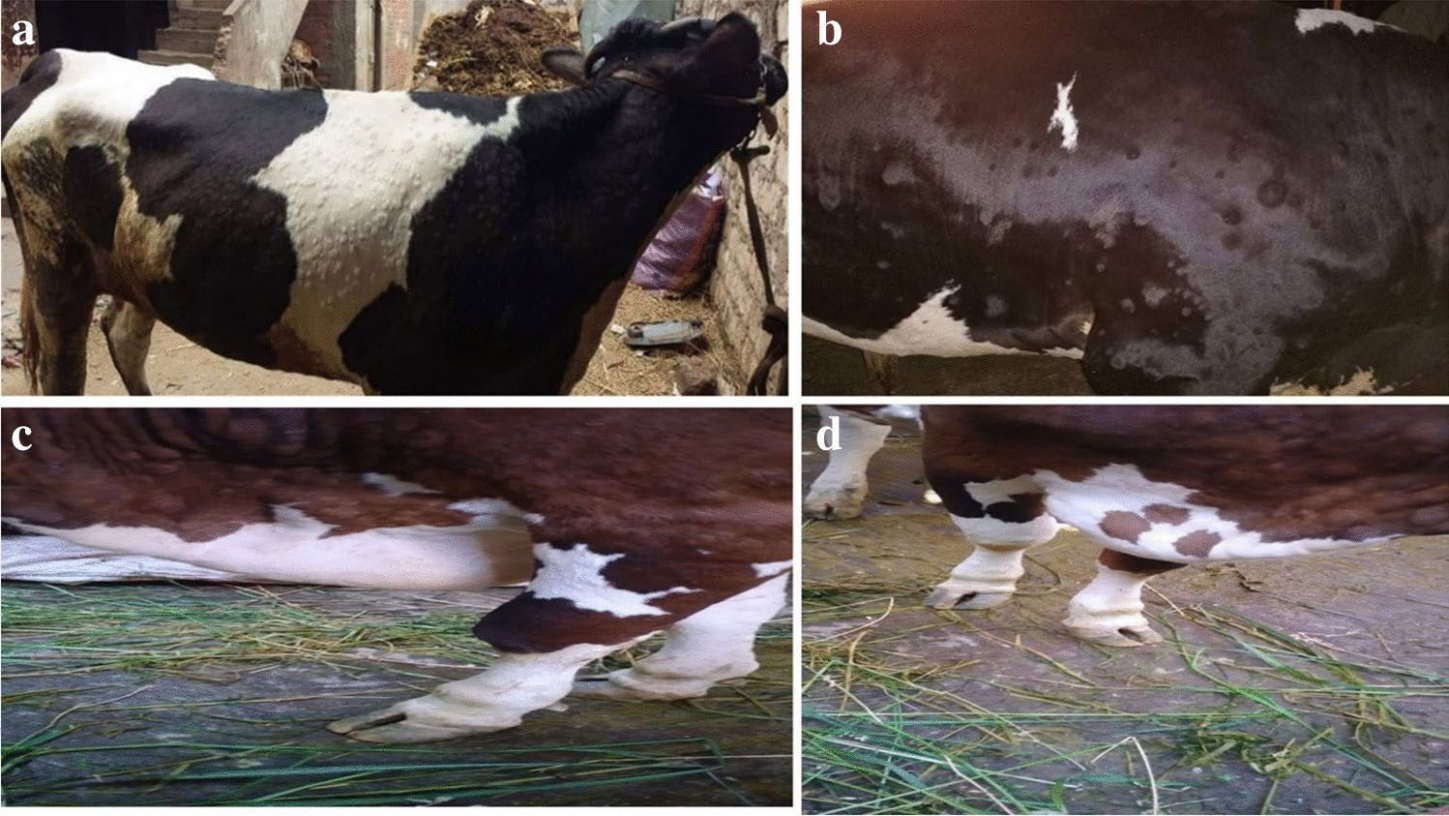
Shows the different stages of lumpy skin disease (LSD) in cattle, with characteristic skin nodules. (**a**) In the early stages of LSD, the cow developed small, raised nodules on its skin (**b**). In the later stages of LSD, the cow may develop larger, more widespread nodules. These Nodules may be up to 5 cm in diameter and may be very painful. (**c**) In severe cases the cow develops edema, or swelling, in its dewlaps. (**d**) The severity of the illness: Cows may develop severe edema in dewlaps with ulceration of the hoof

The animals exhibited an increase in body temperature of 40 to 41.5°C, excessive tearing, a decrease in milk production, anorexia, a loss of weight, and various stages of firm skin nodules all over the body of the infected animals. Nodules are raised, circumscribed, firm, and accompanied by swelling of the superficial lymph nodes. as shown in Table 3, Figs. 1 and 2. The different stages of lumpy skin disease (LSD) in cattle with characteristic skin nodules. Figure 2 (a) shows that in the early stages of LSD, the cow developed small, raised nodules on its skin. Figure 2 (b) shows that in later stages of LSD, the cow may develop larger, more widespread nodules. These nodules may be up to 5 centimeters in diameter and may be very painful. Figure 2 (c) shows that in severe cases, the cow develops edema or swelling in its dewlaps. Figure 2 (d) shows the severity of the illness: Cows may develop severe edema in dewlaps with ulceration of the hoof.

**Table 3:** Collection of Ticks from Cattle and Buffaloes with Clinical Signs of Lumpy Skin Disease (LSD) or without any Lumpy Skin Disease signs (WLSD) in Different Locations or Governorates

Location or Governorates	T	Cattle		T	Buffaloes		T	Ticks from cattle		T	Ticks from buffaloes	
		LSD	WLSD		MLSD	WLSD		LSD	WLSD		LSD	WLSD
Beni-suef	20	6	14	5	2	3	25	15	10	11	5	6
El Fayoum	15	5	10	10	3	7	17	12	5	10	4	6
Giza	13	5	8	10	4	6	18	13	5	4	4	-
Monefia	3	2	1	2	1	1	13	10	3	6	6	-
Sharqiya	16	7	9	7	3	4	13	3	10	3	3	-
Marsa Matrouh	3	2	1	2	1	1	6	6	-	6	2	4
Gharbiya	14	3	11	9	4	5	9	5	4	4	4	-
Total	84	30	54	45	18	27	101	64	37	44	28	16

T: total number of cattle or buffaloes, LSD: Lumpy Skin Disease, MLSD: mild clinical Lumpy Skin Disease signs

WLSD: without any Lumpy Skin Disease signs

### Identification of tick species

**Fig. 3:**
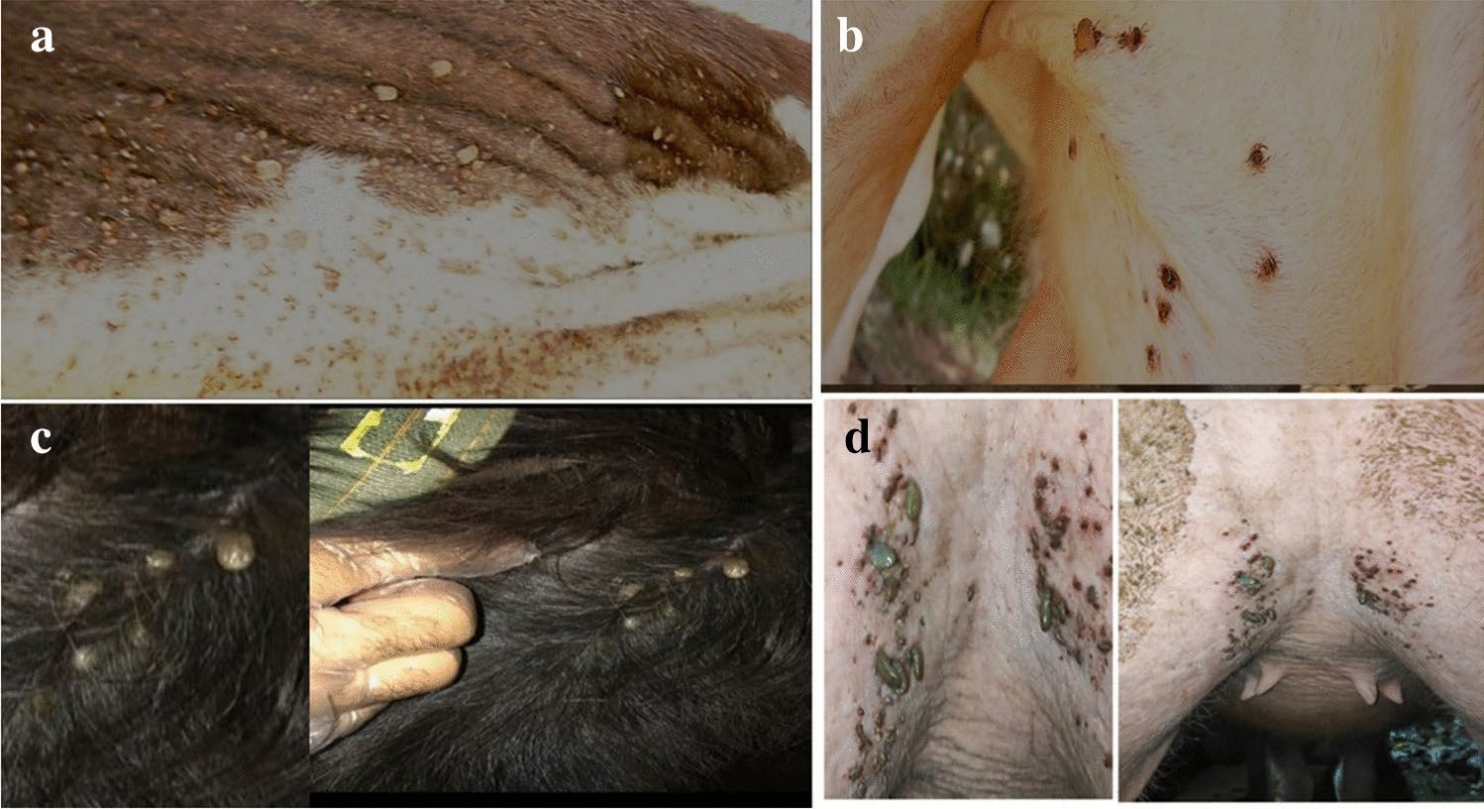
Cattle infested by tick species. **a** and **b**) A heavy infestation of the animal body by the *Rhipicephalus (Boophilus) annulatus* tick. The ticks are engorged with blood and are visible on the cattle's back, neck, and legs. (**b**) A slight infestation of the animal body *R. (B.) annulatus* ticks. They are mostly found on the cattle's head and neck. **c**, and **d** hard tick infested buffaloes’ adults and calve

Figure 3 shows ticks collected from different animals (cattle and buffaloes) with or without LSD clinical signs. Morphological identification revealed that they belonged to *R. (Boophilus) annulatus*, the most prevalent tick species on both cattle and buffaloes. Their dorsal shield (*scutum*) and forward-protruding mouthparts (capitulum) were observed. It belongs to the *Boophilus* subgenus and exhibits a hexagonal basis capitulum with rounded or oval spiracular plates and very short, compressed palps featuring dorsal and lateral ridges. Male hard ticks possess adanal shields and accessory shields, while females typically lack a distinct anal groove, which is faintly present in males. There are no evident festoons or ornamentation.

Table 4 presents the results of the detection of LSDV antibodies and LSDV-DNA in cattle and buffaloes' sera, as well as whole blood samples, using EP-ELISA and PCR across different governorates of Egypt. Out of 84 sera samples, 46 (54.8%) tested positive for LSDV antibodies by EP-ELISA in both vaccinated and non-vaccinated cattle. While PCR detected LSDV-DNA in 30 out of 84 (35.7%) whole blood samples obtained from cattle, In the case of buffaloes, antibodies against LSDV were 15 positives out of 45 (33.3%) detected by EP-ELISA. Additionally, PCR identified LSDV-DNA in 5 out of 45 (11.1%) buffaloes. The highlights highlight variations in LSDV prevalence across different governorates, with the highest percentage of active LSDV infections found in El-Fayoum (46.6%), followed by Sharqiya (37.5%), and Monefia (33.3%). Conversely, the lowest percentage of LSDV-positive samples was observed in Giza (7.6%). The results showed that cattle are more likely to test positive for LSDV compared to buffaloes.

**Table 4:** LSDV was detected in blood samples from examined animals using end-point ELISA and PCR for identification of LSDV antibodies and LSDV-DNA in cattle and buffaloes in different governorates in Egypt. Cattle exhibited positive results for LSDV antibodies through EP-ELISA of 54.8%, while 35.7% were positive by PCR. Similarly, buffaloes positive for LSDV antibodies using EPELISA were 33.3 but 11.11% positive by PCR

Governorates	Cattle					Buffalos				
	Total No	Indirect EP-ELISA sera		PCR whole blood EDTA		Total No	Indirect EP-ELISA sera		PCR whole blood EDTA	
		+ ve	%	+	%		+ ve	%	+ ve	%
Beni-suef	20	12	60	5	25	5	2	40	1	20
El Fayoum	15	8	53.3	7	46.6	10	2	20	1	10
Giza	13	6	46.1	3	7.6	10	3	30	1	33.3
Monefia	6	2	33.3	2	33.3	2	-	-	-	-
Sharqiya	16	9	56.2	9	37.5	7	3	42.8	1	14.28
Marsa Matrouh	5	3	60	1	33.3	2	-	1-	-	-
Gharbiya	10	6	60	5	21.4	9	5	55.5	1	11.11
Total	84	46	54.8	30	35.7	45	15	33.3	5	11.11

EP-ELISA: End point—Enzyme-Linked Immunosorbent Assay; PCR: Polymerase Chain Reaction

### Virus isolations

**Fig. 4:**
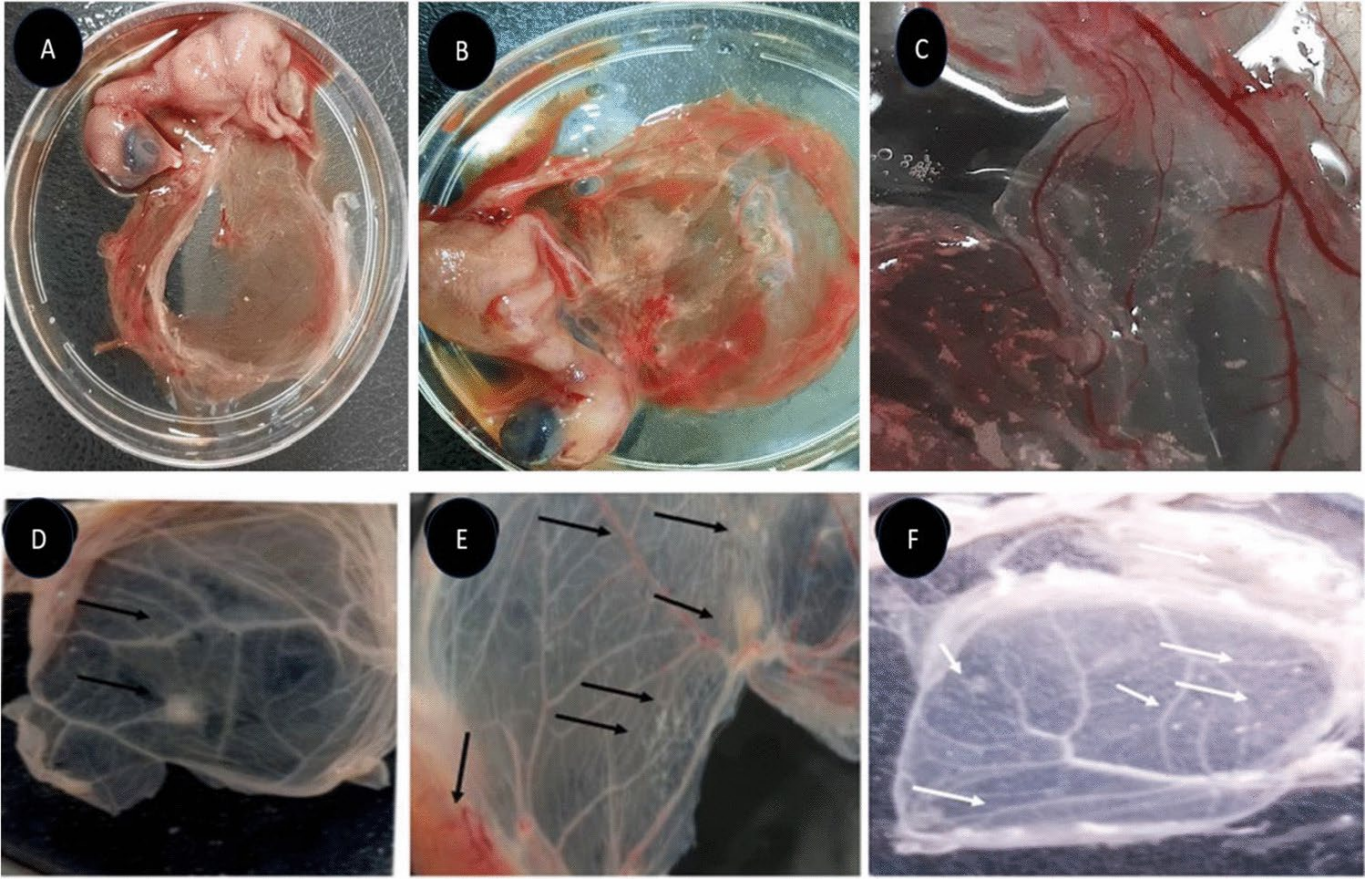
**a-f** showed CAM thickening of the membrane. Figure 4d-f: Characteristic pock lesions of LSDV isolated from homogenates of skin biopsies and ticks’ samples in CAM were observed

Isolation of LSDV from tick samples and inoculation into SPF ECE, as shown in Fig. 3, three pools each (3 to 5 ticks) of collected different ticks’ stages (adults and nymphs) from clinically suspected LSDV-infected cattle gave a positive result in rt-qPCR. By the third passage, three pooled samples were found to be positive out of 29 pooled samples. Harvested CAM showed pock lesions and thickening of the membrane, as shown in Fig. 4a–f. The pock lesions in Fig. 4b are likely caused by the virus destroying the cells of the CAM.

### Characterization of an isolated virus and virus detection

#### Multiplex PCR

**Fig. 5:**
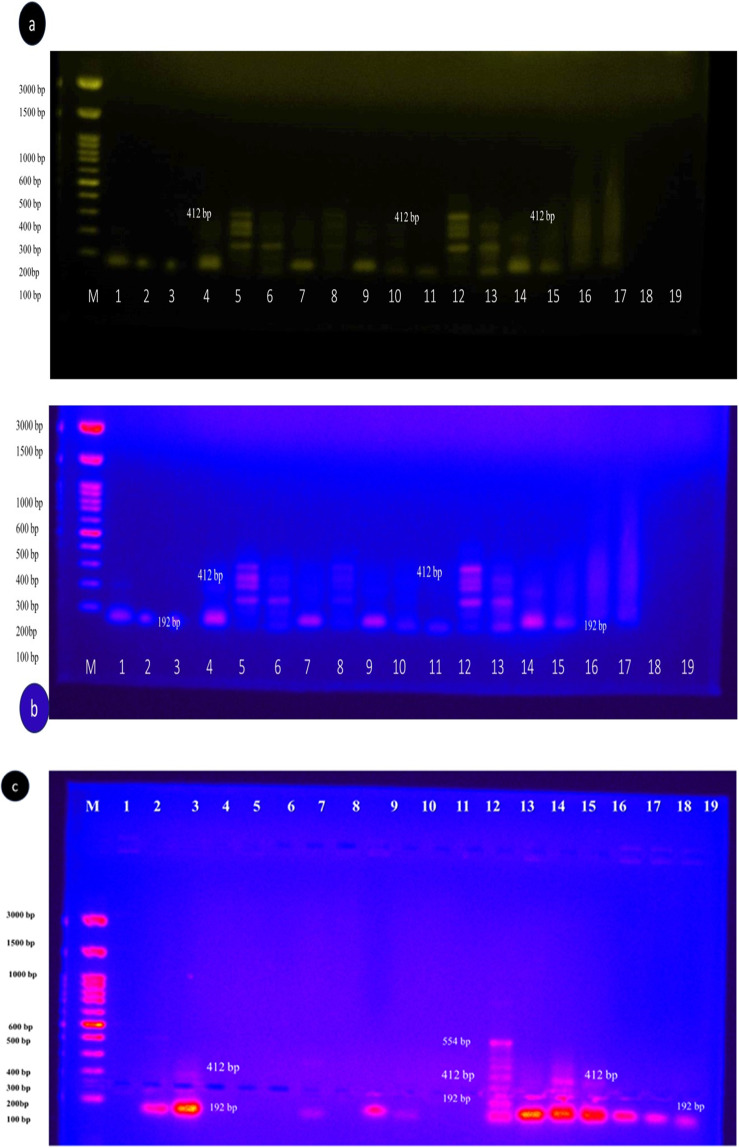
(**a**) Multiplex PCR for amplified products of LSDV isolated from skin biopsy, blood, and ticks' samples using unique primers for P32, VP32, G protein, and viral fusion protein genes. Lane M: Marker with molecular weight marker (3000 bp), Lane 2, 3, 11, 15, 16, 17, 18 and19; Negative results Lane 4: Positive 192 bp, Lane 6, 8,10, 12 and 13: Positive at 192 bp, 412 bp, and 554 bp Lane 9; Fint positive. Lane 1C-: Negative Control; Lane 5 C + : Positive control for LSDV. (**b**) Multiplex PCR for amplified products of LSDV isolated from skin biopsy, blood, and ticks' samples using unique primers for P32, VP32, G protein, and viral fusion protein genes. Lane M: Marker with molecular weight marker (3000 bp), Lane 5 and 12 positive results at 412 bp Lane 1,2,3,4,6,7,9,11,12,13,14, and 15 positive at 192 bp, Lane 1C-: Negative Control; Lane 5 C + : Positive control for LSDV. (**c**) Multiplex PCR for amplified products of LSDV isolated from skin biopsy, blood, and ticks' samples using unique primers for P32, VP32, G protein, and viral fusion protein genes. Lane M: Marker with molecular weight marker (3000 bp), Lane 2,3,6,8,9, 12 13, 14,15,16,17, and 18 positives at 192 bp, Lane 12 and 14: positive at 192 bp, 412 bp, and 554 bp Lane 9; Fint positive, Lane 1, 4, 5,6,8,10,11 and 19 negative results

For this study, a multiplex PCR assay was used to amplify LSDV-DNA from skin, blood, and tick samples, using specific primers for P32, VP32, G protein, and viral fusion protein genes. Gel electrophoresis (Fig. 5a, b, and c) revealed that the positive LSDV results : in Fig. 5a, Lane 4 showed a 192 bp positive result; in Fig. 5b, Lanes 5 and 12 were positive at 412 bp, and Lanes 1, 2, 3, 4, 6, 7, 9, 11, 12, 13, 14, and 15 showed a 192 bp positive band; in Fig. 5c, Lanes 2, 3, 6, 8, 9, 12, 13, 14, 15, 16, 17, and 18 displayed a 192 bp positive band, while Lanes 12 and 14 exhibited bands at 192 bp, 412 bp, and 554 bp. Table 5 shows positive detections of LSDV-DNA in ticks from cattle and buffaloes and non-LSD samples across various stages of ticks (adults, nymphs, eggs, and larvae) concerning transmission of LSDV through different stages. The positive rates of engorged female and nymph ticks from cattle with or without LSD and the numbers of eggs and larvae collected from both LSD-infected and non-LSD-infected cattle provide insight into the detection rates of LSDV in different stages of ticking under varying conditions.

**Table 5:** Detection of LSDV in ticks’ samples collected from different animals by RT-qPCR and PCR. Detections of LSDV-DNA in ticks, from cattle and buffaloes and non-LSD samples through different stages of ticks (adults, nymphs, eggs, and larvae). The positive rates of engorged female and nymph ticks from infected and non-LSD-infected cattle and buffaloes

Groups of Ticks	Detection LSDV-DNA in ticks by	PCR detected LSDV-DNA in ticks collected from					rt-qPCR Syber Green based detected LSDV-DNA in ticks collected from			
		Cattle			Buffaloes		Cattle		Buffaloes	
		+ *ve*			+ ve		+ ve		+ ve	
Ticks Adults and nymph	LSD 8 pools *	5	+		2	+	7	+	2	+
	Non-LSD 6 pools**	3	+		1	+	5	+	1	+
Eggs	LSD 8 ***	5	+		2	+	7	+	2	+
	Non-LSD 6****	3	+		1	+	5	+	1	+
Larvae	LSD 8*****	5	+		2	+	7	+	2	+
	Non-LSD 6*****	3	+		1	+	5	+	1	+

#### Real-time quantitative PCR (rt-qPCR)

Detection of LSDV from suspected animal samples in different governorates in Egypt by rt-qPCR. The results revealed varied percentages of LSDV-positive samples across different regions. The results showed that the percentage of LSDV-positive samples was in El-Fayoum (46.6%), followed by Sharqiya (37.5%) and Monefia (33.3%) of blood samples. The lowest percentage of LSDV-positive samples was in Gharbiya (21.4%). In total, 30 cattle skin biopsy samples were tested by rt-qPCR, of which 15 were positive for LSDV (50%). Only five blood samples from buffaloes were positive for LSDV (11.11%). The Ct values in the table can be used to estimate the amount of LSDV DNA in the samples, as shown in Table 6. The amplification of target DNA was detected using fluorescent molecules, and the cycle threshold (Cq) number of amplifications of positive samples ranged from 20 to 21 (Fig. 4). The melting curve and melting peak for the envelope protein (P32) gene in LSDV-positive samples were calculated using a 2x SYBR Green DNA polymerase master mix, and the chart was generated by plotting relative fluorescence against cycle number.

**Table 6:** Detection of LSDV from suspected cattle samples by molecular assays (rt-qPCR and PCR), the highest percentage of LSDV-positive samples was in Beni-suef (66.6%). The Ct values in this study range from 10 to 36.5 proved that the amount of LSDV DNA in the samples varied

Governorates	Whole blood	Nasal samples	Skin biopsy	PCR detected LSDV_DNA from different samples						rt-qPC detected LSDV-DNA from different samples					
				Whole blood		Nasal samples		Skin biopsy		Whole blood		Nasal samples		Skin biopsy	
				+	%	+	%	+	%	+	%	+	%	+	%
Beni-suef	20	10	6	5	25	2	20	2	33.3	8	40	3	30	4	66.6
El Fayoum	15	15	5	7	46.6	2	13.3	1	50	7	46.6	3	20	1	50
Giza	13	10	5	3	7.6	1	16.6	2	33.3	3	7.6	1	16.6	2	40
Monefia	3	3	2	2	33.3	-		3	75	2	33.3	-		3	75
Sharqiya	16	16	7	9	35.7	3	18.75	3	42	9	37.5	3	18.75	3	42
Marsa Matrouh	3	2	2	1	33.3	-		1	33.3	1	33.3	-		1	33.3
Gharbiya	14	14	3	5	21.4	2	14.28	3	75	5	21.4	2	14.28	3	75
Total	84	70	30	30	23.2	10	14.2	15	50	35	42.6	12	17.1	17	56.6

#### Ct-value

The Ct values in this study ranged from 20.9 to 36.5, suggesting that the amount of LSDV DNA in the samples varied, (with excluded Ct 10.2) as shown in Table 6 showed rt-qPCR Syber Green, fluorescence development over time (graph generated by ES Equant tube scanner software) for detection of LSDV-DNA in skin tissue biopsy of infected cattle and buffaloes; Fig. 5b: Detection of LSDV-DNA in blood and nasal swabs showed different results. The rt-qPCR amplification plot with Syber Green A green dye showed positive results with the positive control of the sheep pox virus vaccine. Figure 5c: rt-q PCR amplification plot of tick samples and LSDV isolated on CAM were compared with the positive control of Neethling virus. Figure 5d rt-q PCR amplification plot with TaqMan probe for detection of LSDV-DNA in skin biopsies and tick samples compared with control positive and other results from rt-q PCR Syber Green. The Ct value of biopsy samples (red square) was the highest, followed by swab samples (gray triangle). The Ct values of the blood sample were comparable to those of biopsy and other samples, Ct ranged from 10.2 to 36.6. This variation indicates differing levels of LSDV-DNA across samples. Biopsy samples were found to be the most reliable for detecting LSDV-DNA, though swab and blood samples also showed potential. Ct values suggest varying DNA quantities influenced by sample type and infection severity (Fig. 8). Lower Ct values indicate higher LSDV DNA presence, spanning 10.2 to 36.6 in this study, highlighting sample-specific variations.

## Discussion

Despite extensive vaccination efforts using heterologous vaccines that have shown some success in controlling LSDV outbreaks in Egypt, outbreaks continue to cause substantial economic losses in the livestock sector. However, Neethling-based LSD vaccines, while offering promise, have shown drawbacks such as severe post-vaccination reactions and limited effectiveness. So, the consequences of LDS outbreaks are not directly related to the extensive vaccination efforts (Akther et al. 2023). Partial protection against LSD in cattle has been achieved in Egypt with an attenuated *Romanian strain* of SPV vaccine (Gaber et al. 2022). The clinical observations were supported by previous studies and emphasize characteristic signs of LSD, including fever, increased nasal secretions, salivation, skin nodules, and enlarged superficial lymph nodes, as shown in Figs. 1 and 2 and Table 3. While LSD primarily affects cattle, our observations, along with previous research, suggest that water buffaloes may serve as accidental hosts infected with LSDV or may be resistant to infection with LSDV, exhibiting fewer clinical signs compared to infected cattle (Zeedan et al. 2019; Khan et al. 2021; Amin et al. 2021; Selim et al. 2021), and this observation was agreed with (Sharawi and Abd El-Rahim 2011; El-Tholoth and El-Kenawy 2016). They found water buffaloes showed LSD signs, and their role in the epidemiology of LSD remains unclear in Egypt. Evidence of a natural infection with LSDV in buffalo has been reported in Egypt (El-Tholoth and El-Kenawy 2016). In the present study, clinical examination of different animals was supported by laboratory diagnostic tests, including EP-ELISA and PCR, as shown in Table 4, which detected LSDV antibodies and DNA in serum and whole blood samples from examined cattle and buffaloes in different governorates. Specifically, in cattle, 60% tested positive for LSDV antibodies by EP-ELISA, while 30% were positive for LSDV DNA by mPCR. Although, in buffaloes, 40% tested positive for LSDV antibodies by EP-ELISA and 20% were positive for LSDV DNA by mPCR, these results agree with those from Wang et al. (2021). The findings also indicate that ELISA is a valuable diagnostic tool for LSD seroprevalence studies (OIE 2010). In a previous study (Fagbo et al. 2014) indicated that 5 of 66 buffaloes tested positive for LSDV antibodies with low titers using a serum neutralization. In another study (Barnard 1997), no LSDV antibodies were detected in 15 buffaloes from South Africa.

The findings in Fig. 4 and Tables 5 and 6 showed viral isolation on CAM and identification using multiplex gel-based PCR, which confirmed LSDV as the causative agent of outbreaks in Egypt. These results agreed with El-Kenawy and El-Tholoth (2010). The positive result was noted in different regions, with Beni-Suef, Sharqiya, and Gharbiya showing similar prevalence rates of 60%, followed by El Fayoum at 53.3%. It is worth noting that some animals may be vaccinated or previously infected, potentially leading to an incomplete level of protective immunity against LSDV. This observation agreed with previous studies (Abdallah et al. 2018; Bamouh et al. 2021), which reported that LSDV vaccination resulted in the production of antibodies in 50% of cattle after three weeks, while SPV vaccines failed to elicit an antibody response. The failure of heterologous vaccines may be attributed to LSD signs manifesting in vaccinated animals’ post-vaccination. This highlights the importance of considering vaccination history and previous exposure when interpreting serological test results for LSDV. While multiplex PCR and rt-qPCR are effective in detecting and distinguishing between active infection with LSDV and vaccinated animals, the diagnosis of LSD typically relies on identifying characteristic clinical signs and molecular diagnosis using multiplex PCR and rt-qPCR (Zeedan et al. 2019; Selim et al. 2021; Bianchini et al. 2023). Considering the inability to detect LSDV in buffaloes using PCR and the limited success in detecting antibodies, except in cases with poor seroconversion, it suggests that the Egyptian water buffalo might not be susceptible to LSDV and could potentially serve as an accidental host. However, the low proportion of positive buffalo sera could also be attributed to false positives due to unknown test specificity, which is reinforced by the negative results of skin nodules for LSDV. Furthermore, LSD cases have been exclusively reported in cattle at the Ismailia abattoir in Egypt, with no recorded instances in buffaloes or cattle calves (Ahmed and Dessouki 2013) This explanation disagrees with (El-Nahas et al. 2011, and Sharawi and El-Rahim 2011), who recently documented molecular diagnosis of LSDV in naturally infected water buffalo in Egypt. However, the PCR assays utilized in these studies were unable to differentiate between different *Capripoxviruses*.

**Fig. 6:**
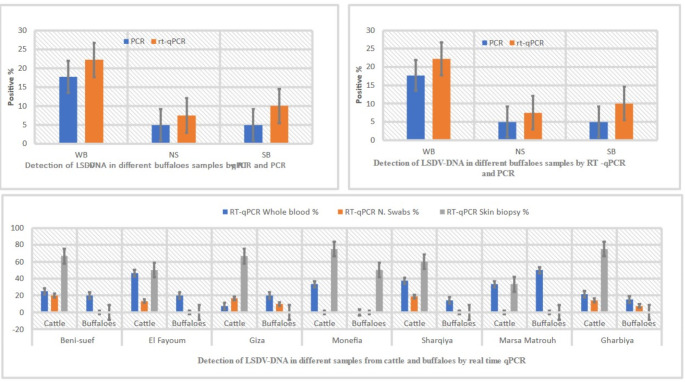
Showed the rt-qPCR and PCR positivity rates of animal samples in Egyptian Governorates, the positivity rates obtained through PCR and rt-qPCR tests conducted on samples from cattle and buffaloes in different governorates in Egypt for different samples types (whole blood, nasal swabs, skin biopsy)

Direct or indirect contact remains the primary mode of LSDV transmission, but understanding the potential role of hard ticks is crucial for effective control (Abera et al. 2015; Leal 2018; Renald et al. 2023). The transmission of LSDV involves both mechanical and biological routes, with environmental factors such as the presence of watercourses and ponds near cattle populations contributing to increased insect vector abundance. In the present study, cattle exhibited moderate to heavy infestations by hard ticks, indicating their potential involvement in LSDV transmission, as shown in Figs 2 and 3. Engorged female ticks from both cattle and buffaloes carried LSDV, even in animals without clinical signs of LSD. Our experiment's detection of LSDV-DNA in ticks in different stages and experimental conditions sheds light on the potential transmission of LSDV through various tick life stages, as shown in Scheme 1 and Table 5. The experimental conditions, support the hypothesis that ticks play a role in LSDV transmission (Sanz-Bernardo et al. 2021; Sanz-Bernardo et al. 2022; Sariya et al. 2022). Therefore, in light of the evidence presented in the manuscript, it is reasonable to acknowledge the potential role of ticks in LSD spread. The references provided to studies such as Tuppurainen et al. (2013). *Rhipicephalus annulatus,* found on both cattle and buffaloes in subtropical and tropical regions, is considered a potential carrier of LSDV due to its widespread distribution in Egypt. The tick homogenates and skin biopsies from diseased and asymptotic cattle and buffaloes induced pock lesions on CAMs, as shown in Fig. 4, and were confirmed by mPCR and rt-qPCR as shown in Figs. 5 and 6. This isolation of the virus from ticks supports their potential role in LSDV transmission; these findings were agreed upon (Tuppurainen et al. 2013; Tuppurainen et al. 2015, 2017; El-Ansary et al. 2022). Previous studies have shown LSDV survival in ticks, even without LSD symptoms, while ticks feeding on viremic cattle tested positive (Sprygin et al. 2019; Sudhakar et al. 2022; Cook et al. 2023; Saleh et al. 2023). Also supported by reports of vertical transmission through infected *R. decoloratus* females to bovine hosts (Perveen et al. 2021; Saleh et al. 2023; Ravindran et al. 2023). *R. annulatus* ticks, capable of surviving without feeding for extended periods and completing multiple generations per year, may serve as reservoir hosts for LSDV, contributing to its widespread nature in different governorates of Egypt.

**Fig. 7:**
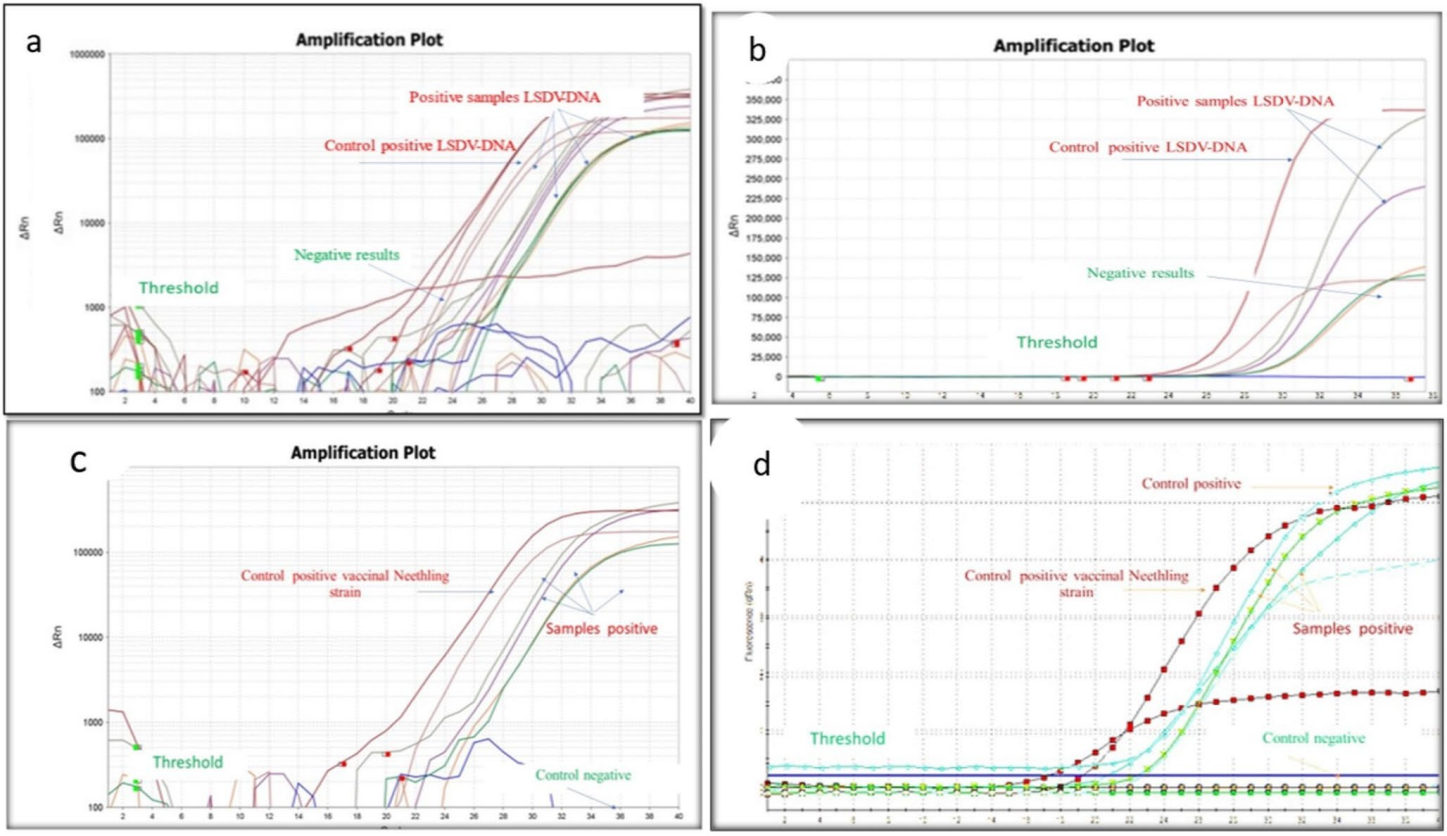
**a** Real-time quantitative PCR (rt-qPCR) amplification plot with Syber Green that showed fluorescence development over time (graph generated by ES Equant tube scanner software) for detection of LSDV-DNA in skin tissue biopsy of infected cattle and buffaloes; Fig. 6a showed the real-time quantitative PCR (rt-qPCR) amplification plot using Syber Green, demonstrating the gradual development of fluorescence over time. This graph, generated by the ES Equant tube scanner software, illustrates the detection of LSDV-DNA in skin tissue biopsy samples obtained from infected cattle and buffaloes. **b**, the detection of LSDV-DNA in blood and nasal swabs revealed that the rt-qPCR amplification plot utilizing Syber Green Dye exhibits positive results, notably with the positive control of Neethling virus vaccine. **c** showed the rt-qPCR amplification plot using Syber Green Dye, comparing ticks' samples and LSDV isolated on CAM (chorioallantoic membrane) with the positive control of Neethling virus. **d**, the rt-qPCR amplification plot utilizing TaqMan probe for LSDV-DNA detection in skin biopsies and ticks’ samples compared to the positive control and other results obtained from rt-qPCR using Syber Green

When comparing the TaqMan rt-qPCR probe and Syber Green, both methods exhibited nearly identical results for detecting LSDV-DNA, as illustrated in Figs. 6 and 7. This underscores the consistency and reliability of both techniques in detecting LSDV, offering flexibility in choosing the appropriate method for specific laboratory settings.

A real-time PCR assay detected LSDV-DNA in biopsy samples with the lowest Ct values, making them most suitable for testing. The rt-qPCR assay detected LSDV-DNA in 35 whole blood samples, 12 nasal swabs, and 17 skin biopsies more than conventional gel-based mPCR, which detected only 30, 10, and 15 in blood EDTA, nasal swabs, and skin biopsies, respectively, as shown in Tables 6 and 7. The lower incidence of LSDV infection in some governorates may be attributed to the predominant rearing of buffaloes, which are known for their resistance to LSDV. Alternatively, it could be indicative of a lower manifestation of ticks, as suggested by previous studies (Elhaig et al. 2017; Akther et al. 2023).

**Table 7:** Detection of LSDV from suspected buffalos’ samples by molecular assays (rt-qPCR and P CR). Buffaloes in Beni-suef, El Fayoum, and Giza governorates showed a higher number of positive LSDV-DNA across various sample types. While, Monefia had a significant percentage of positive skin biopsy samples

Governorates	WB	NS	SB	PCR						RT-qPCR		qPCR			
				WB*		NS**		SB***		WB*		S**		SB***	
				+	%	+	%	+	%	+	%	+	%	+	%
Beni-suef	5	5	3	1	20	-	-	0	0	1	20	-	-	0	0
El Fayoum	10	10	3	2	20	-	-	0	0	2	20	-	-	0	0
Giza	10	10	4	2	20	1	10	0	0	2	20	1	10	0	0
Monefia	2	2	1	-	-	-		1	50	-	-	-		1	50
Sharqiya	7	7	3	1	14.2	-	-	0	0	1	14.2	-	-	0	0
Marsa Matrouh	2	1	1	1	50	-		-	-	1	50	-		-	-
Gharbiya	9	9	5	2	15.4	1	7.6	0	0	2	15.4	1	7.6	0	0
Total	45	44	20	9	20	2	4.54	1	5	9	20	2	4.54	1	5

WB*: Whole blood

NS**: Nasal samples

SB***: Skin biopsy

The Ct (cyclic threshold) values obtained in this study provide insights into the quantity of LSDV-DNA in different samples, indicating potential variations based on the sample nature and the severity of the infection. This finding was in agreement with previous studies (Lubinga et al. 2015; Zeedan et al. 2019). The Ct values analyzed in skin biopsies, swabs, and blood samples collected from both cattle and buffaloes suggested that skin biopsy samples offer the highest sensitivity for detecting LSDV-DNA.

**Fig. 8:**
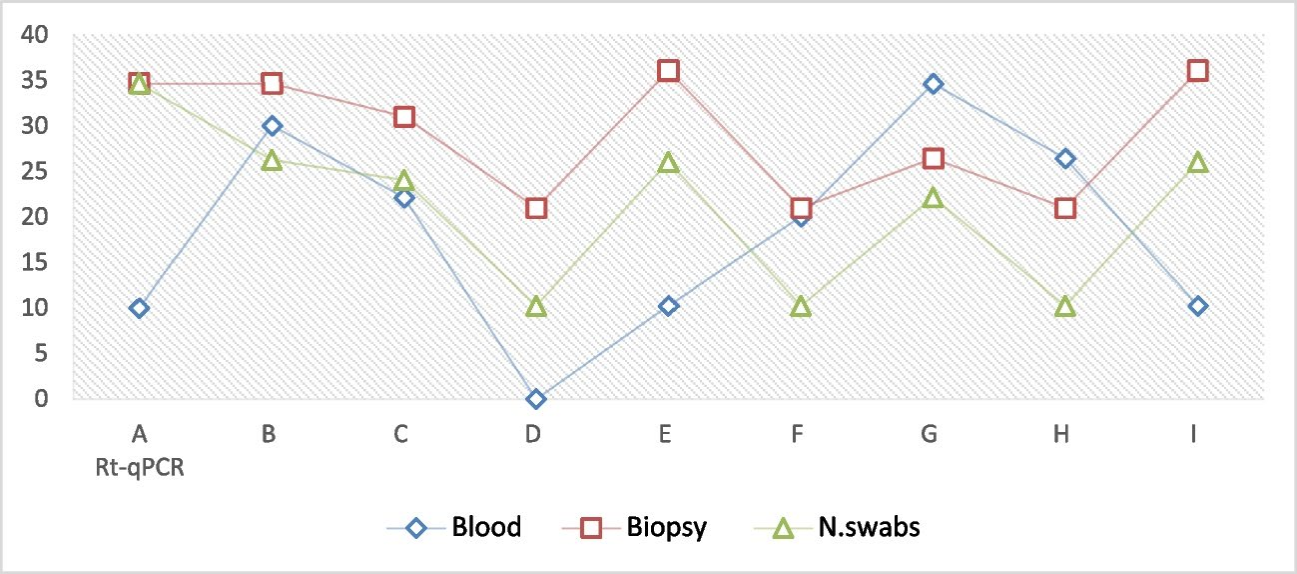
Ct-Value distribution in 19 scab, swab, and blood-positive samples for LSDV-DNA from cattle and buffaloes. Ct value of biopsy samples (Red Square) was the highest, follow by swab sample (Geen Tringale). While, Ct value of blood sample (blue Diamond shape) was similar to that of biopsy samples and other types of samples., Ct values span a range from 10.2 to 36.6, indicating variations in viral DNA abundance across samples. The outcomes of this investigation propose that biopsy samples serve as the most reliable source of LSDV-DNA. Nonetheless, swab samples and blood samples can also be employed for detection. It is evident from the Ct values in this study that the quantity of LSDV-DNA

However, the study also indicates that swabs and blood samples can be viable alternatives, providing flexibility in sample collection methods, as illustrated in Fig. 8. Furthermore, the real-time PCR assay employed in this study demonstrated a high level of specificity with no cross-reaction observed, confirming its reliability for differentiating the virus from other potential genetic material present in the samples (Mashamba 2020; Abdalhamed et al. 2022). This study showed the positive result of LSDV infection in blood samples and hard ticks from buffalo’s samples, suggesting buffaloes and hard ticks may play a role in LSDV transmission in Egypt, but large-scale molecular surveillance is urgently needed to determine their susceptibility to LSDV infection.

## Conclusion

Lumpy skin disease virus is a transboundary re-emerging disease with highly significant economic losses. Our study confirmed that LSDV is currently circulating in Egypt, despite regular vaccination programs using sheep pox vaccines. The positive results of multiplex PCR and rt-qPCR assays on *Rhipicephalus annulatus* ticks proved that ticks can serve as a natural reservoir host for LSDV. Additionally, the mild clinical symptoms and few positive samples collected from buffaloes revealed that buffaloes may be accidental, non-adapted hosts. rt-qPCR and multiplex PCR are needed for rapid routine diagnostic assays for LSDV from different samples. The findings of this study will help inform the development of more effective control strategies for this disease.

## Supplementary information

Below is the link to the electronic supplementary material.

Supplementary file1 (JPG 67 KB) **Supplementary Figures   **Figs Multiplex PCRfor amplified products of LSDV isolated from skin biopsy, blood, and ticks'samples using unique primers for P32, VP32, G protein, and viral fusion proteingenes. Lane M:   Marker with molecular weightmarker (1200-3000 bp), Positive at 192bp, 412 bp, and 554 bp beside NegativeControl; and Positive control for LSDV.

## Data Availability

All data generated or analyzed during this study are available via contact with Dr. Gamil SG Zeedan and/or Dr. Abeer M Abdalhamed who are colleagues of the study and co-authors. they emails are: gs.zeedan@nrc.sci.eg and am.abdelhamed@nrc.sci.eg

## References

[CR1] Abdalhamed AM, Naser SM, Mohamed AH, Zeedan GS (2022) Development of gold nanoparticles-lateral flow test as a novel field diagnostic assay for detecting foot-and-mouth disease and lumpy skin disease viruses. Iran J Microbial 14:574–586. 10.18502/ijm.v14i4.10245 10.18502/ijm.v14i4.10245 PMC9867639 36721504

[CR2] Abdallah FM, El Damaty HM, Kotb GF (2018) Sporadic cases of lumpy skin disease among cattle in Sharkia province, Egypt: genetic characterization of lumpy skin disease virus isolates and pathological findings. Vet World 11:1150–1158. 10.14202/vetworld.2018.1150-1158 30250377 10.14202/vetworld.2018.1150-1158 PMC6141277

[CR3] Abebe WM (2018) Bovine lumpy skin disease: epidemiology, economic impact and control opportunities in Ethiopia. Wageningen Univ Res. 10.18174/430314

[CR4] Abera Z, Degefu H, Gari G, Ayana Z (2015) Review on epidemiology and economic importance of lumpy skin disease. Int J Basic Appl Virol 4:08–21. 10.5829/idosi.ijbav.2015.4.1.9117

[CR5] Ahmed A, Dessouki A (2013) Abattoir-based survey and histopathological findings of lumpy skin disease in cattle at Ismailia Abattoir. Int J Biosci Biochem Bioinformatics 3:372–375

[CR6] Ahmed EM, Eltarabilli MM, Shahein MA, Fawzy M (2021) Lumpy skin disease outbreaks investigation in Egyptian cattle and buffaloes: serological evidence and molecular characterization of genome termini. Comp Immunol Microbiol Infect Dis 1:76–101. 10.1016/j.cimid.2021.101639 10.1016/j.cimid.2021.101639 33770551

[CR7] Akther M, Akter SH, Sarker S, Aleri JW, Annandale H, Abraham S, Uddin JM (2023) Global burden of lumpy skin disease, outbreaks, and future challenges. Viruses 15:1861–1891. 10.3390/v15091861 37766268 10.3390/v15091861 PMC10535115

[CR8] Aleksandr K, Olga B, David WB, Pavel P, Yana P, Svetlana K, Alexander N, Vladimir R, Dmitriy L, Alexander S (2020) Non-vector-borne transmission of lumpy skin disease virus. Sci Rep 10:7436. 10.1038/s41598-020-64029-w 32366872 10.1038/s41598-020-64029-w PMC7198617

[CR9] Amin DM, Shehab G, Emran R, Hassanien RT, Alagmy GN, Hagag NM, Abd-El-Moniem MI, Habashi AR, Ibraheem EM, Shahein MA (2021) Diagnosis of naturally occurring lumpy skin disease virus infection in cattle using virological, molecular, and immunohistopathological assays. Vet World 14:2230–2237. 10.14202/vetworld.2021.2230-2237 34566343 10.14202/vetworld.2021.2230-2237 PMC8448636

[CR10] Awad WS, Ibrahim AK, Mahran K, Fararh KM, Abdel Moniem MI (2010) Evaluation of different diagnostic methods for diagnosis of Lumpy skin disease in cows. Trop Anim Health Prod 42:777–783. 10.1007/s11250-009-9486-5 19882228 10.1007/s11250-009-9486-5

[CR11] Azeem S, Sharma B, Shabir S, Akbar H, Venter E (2022) Lumpy skin disease is expanding its geographic range: a challenge for Asian livestock management and food security. Vet J 1:279–286. 10.1016/j.tvjl.2021.105785 10.1016/j.tvjl.2021.105785 34915159

[CR12] Bamouh Z, Hamdi J, Fellahi S, Khayi S, Jazouli M, Tadlaoui KO, Fihri OF, Tuppurainen E, Elharrak M (2021) Investigation of post vaccination reactions of two live attenuated vaccines against lumpy skin disease of cattle. Vaccines 9:621–635. 10.3390/vaccines9060621 10.3390/vaccines9060621 PMC8226854 34201339

[CR13] Barnard BJH (1997) Antibodies against some viruses of domestic animals in South African wild animals. Onderstepoort J Vet Res 64:95–110 9352558

[CR14] Bazid AH, Wasfy M, Fawzy M, Nayel M, Abdelmegeid M, Thabet RY, Yong HS, El-Sayed MM, Magouz A, Badr Y (2023) Emergency vaccination of cattle against lumpy skin disease: evaluation of safety, efficacy, and potency of MEVAC® LSD vaccine containing Neethling strain. Vet Res Commun 47:767–777. 10.1007/s11259-022-10037-2 36460903 10.1007/s11259-022-10037-2 PMC9734455

[CR15] Bianchini J, Simons X, Humblet MF et al (2023) Lumpy skin disease: a systematic review of mode of transmission, risk of emergence and risk entry pathway. Viruses 15(1–71):1622. 10.3390/v15081622 37631965 10.3390/v15081622 PMC10458895

[CR16] Buller RM, Arif BM, Black DN, Dumbell KR, Esposito JJ, Lefkowitz EJ, Tripathy DN (2005) Family poxviridae. In: Fauquet CM, Mayo MA, Maniloff J, Desselberger U, Ball LA (eds) Virus taxonomy: classification and nomenclature of viruses. Eighth report of the international committee on taxonomy of viruses. Elsevier Academic Press, San Diego pp 117–133

[CR17] Castro-Janer E, Rifran L, Piaggio J, Gil A, Miller RJ, Schumaker TT (2009) In vitro tests to establish LC50 and discriminating concentrations for fipronil against Rhipicephalus (Boophilus) microplus (Acari: Ixodidae) and their standardization. Vet Parasitol 26(162):120–128. 10.1016/j.vetpar.2009.02.013 10.1016/j.vetpar.2009.02.013 19278787

[CR18] Cook CG, Munyanduki H, Fay PC et al (2023) The mechanical arthropod vector Stomoxys calcitrans influences the outcome of lumpy skin disease virus infection in cattle. bioRxiv. 10.1101/2023.03.13.532343

[CR19] Diab HM, Ahmed SA, Batiha GE-S, Alkazmi L, El-Zamkan MA (2021) Molecular surveillance of lumpy skin disease outbreak, 2019 in Sohag, Egypt: enzootic potential, phylogenetic assessment and implications on cattle herds health. J Anim Health Prod 9:406–416

[CR20] Dubey A, Ghosh NS, Gupta A, Singh S (2023) A review on current epidemiology and molecular studies of lumpy skin disease virus-an emerging worldwide threat to domestic animals. Med Pharm Allied Sci 12:5635–5643. 10.55522/jmpas.V12I1.4583

[CR21] El-Ansary RE, El-Dabae WH, Bream AS, El Wakil A (2022) Isolation and molecular characterization of lumpy skin disease virus from hard ticks, *Rhipicephalus**(Boophilus)**annulatus* in Egypt. BMC Vet Res 18:302–312. 10.1186/s12917-022-03398-y 35932057 10.1186/s12917-022-03398-y PMC9354321

[CR22] Elhaig MM, Selim A, Mahmoud M (2017) Lumpy skin disease in cattle: frequency of occurrence in a dairy farm and a preliminary assessment of its possible impact on Egyptian buffaloes. Onderstepoort J Vet Res 84:1–6. https://hdl.handle.net/10520/EJC-72671f238 10.4102/ojvr.v84i1.1393 PMC6238723 28397517

[CR23] El-Kenawy AA, El-Tholoth MS (2010) Sequence analysis of attachment gene of lumpy skin disease and sheep poxviruses. Virol Sin 25:409–416. 10.1007/s12250-010-3150-0 21221919 10.1007/s12250-010-3150-0 PMC8227941

[CR24] El-Nahas EM, El-Habbaa AS, El-Bagoury GF, Radwan MEI (2011) Isolation and identification of lumpy skin disease virus from naturally infected buffaloes at Kaluobia, Egypt. Glob Vet 7:234–247

[CR25] El-Tholoth M, El-Kenawy AA (2016) G-Protein-Coupled Chemokine Receptor Gene in Lumpy Skin Disease Virus Isolates from Cattle and Water Buffalo (Bubalus bubalis) in Egypt. Transbound Emerg Dis 63(6):e288–e295. 10.1111/tbed.12344 25754131 10.1111/tbed.12344

[CR26] Estrada-Peña A, Bouattour A, Camicas JL, Guglielmone A, Horak I, Jongejan F, Latif A, Pegram R, Walker AR (2006) The known distribution and ecological preferences of the tick subgenus Boophilus (Acari: Ixodidae) in Africa and Latin America. Exp Appl Acarol 38:219–235. 10.1007/s10493-006-0003-5 16596355 10.1007/s10493-006-0003-5

[CR27] Fagbo S, Coetzer JA, Venter EH (2014) Seroprevalence of Rift Valley fever and lumpy skin disease in African buffalo (Syncerus caffer) in the Kruger National Park and Hluhluwe-iMfolozi Park, South Africa. J S Afr Vet Assoc 85(1):1–7 10.4102/jsava.v85i1.1075 25686252

[CR28] Fawzi EM, Morsi AM, Abd-Elfatah EB (2022) Molecular diagnosis of three outbreaks during three successive years (2018, 2019, and 2020) of Lumpy skin disease virus in cattle in Sharkia Governorate, Egypt. Open Vet J 12:451–62. https://www.ajol.info/index.php/ovj/article/view/237484 10.5455/OVJ.2022.v12.i4.6 PMC9473367 36118715

[CR29] Gaber A, Rouby S, Elsaied A, El-Sherif A (2022) Assessment of heterologous lumpy skin disease vaccine-induced immunity in pregnant cattle vaccinated at different times of gestation period and their influence on maternally derived antibodies. Vet Immunol Immunopathol 244:110380. 10.1016/j.vetimm.2021.110380 10.1016/j.vetimm.2021.110380 34998109

[CR30] Gammada I, Morshed MM, Rabby TR, Hossain MI (2022) The prevalence of lumpy skin disease in the cattle population: a brief study. Int J Agric Vet Sci 4:55–67. 10.34104/ijavs.022.055067

[CR31] Gehring R, Mochel JP, Schmerold I (2023) Understanding the background and clinical significance of the WHO, WOAH, and EMA classifications of antimicrobials to mitigate antimicrobial resistance. Front Vet Sci 17:1–12. 10.3389/fvets.2023.1153048 10.3389/fvets.2023.1153048 PMC10063873 37008341

[CR32] Hamza KA, Tamer C, Emre OZ, Mitat KU, Selma KA, Cavunt A, Albayrak H (2023) Molecular investigation of the relationship between vector tick and host in Lumpy Skin Disease. Etlik Vet Mikrobiyol Derg 34:11–15. 10.35864/evmd.1244360

[CR33] Hussein HA, Khattab OM, Aly SM, Rohaim MA (2017) Role of ixodid (Hard) tick in the transmission of lumpy skin disease. Hosts Viruses 4(3):46

[CR34] Ireland DC, Binepal YS (1998) Improved detection of capripoxvirus in biopsy samples by PCR. J Virol Methods 74:1–7. 10.1016/s0166-0934(98)00035-4 9763122 10.1016/s0166-0934(98)00035-4

[CR35] Jena BR, Kantale RA, Dash A, Singh P, Basak G, Singh S, Jadhao A (2022) Emergence of lumpy skin disease virus (LSDV) infection in cattle and buffaloes in India. Epidemiology 11:1857–1861 www.thepharmajournal.com

[CR36] Khan YR, Ali A, Hussain K, Ijaz M, Rabbani AH, Khan RL, Abbas SN, Aziz MU, Ghaffar A, Sajid HA (2021) A review: surveillance of lumpy skin disease (LSD) a growing problem in Asia. Microb Pathog 158(1–8):105050. 10.1016/j.micpath.2021.105050 34146642 10.1016/j.micpath.2021.105050

[CR37] Leal B (2018) Questing activity of cattle fever tick larvae, Rhipicephalus (Boophilus) Microplus (Acari: Ixodidae): environmental influences and implications for control in South Texas. The University of Texas Rio Grande Valley. ProQuest Dissertations Publishing. 10824331.

[CR38] Lubinga JC, Tuppurainen ES, Stoltsz WH, Ebersohn K, Coetzer JA, Venter EH (2013) Detection of lumpy skin disease virus in saliva of ticks fed on lumpy skin disease virus-infected cattle. Exp Appl Acarol 61:129–138. 10.1007/s10493-013-9679-5 23456606 10.1007/s10493-013-9679-5

[CR39] Lubinga JC, Tuppurainen ES, Mahlare R, Coetzer JA, Stoltsz WH, Venter EH (2015) Evidence of transstadial and mechanical transmission of lumpy skin disease virus by A mblyomma hebraeum Ticks. Transbound Emerg Dis 62:174–182. 10.1111/tbed.12102 23702314 10.1111/tbed.12102

[CR40] Mashamba PV (2020) Molecular characterization of lumpy skin disease virus in Mahikeng local municipality (Doctoral dissertation, North-West University (South Africa)

[CR41] Morgenstern M, Klement E (2020) The effect of vaccination with live attenuated neethling lumpy skin disease vaccine on milk production and mortality—An analysis of 77 dairy farms in Israel. Vaccines 8(2):324 32575395 10.3390/vaccines8020324 PMC7350216

[CR42] OIE (2010) OIE Manual of diagnostic tests and vaccines for terrestrial animals. Lumpy skin disease. Chapter2.4. 14: 68–778

[CR43] Perveen N, Muzaffar SB, Al-Deeb MA (2021) Ticks and tick-borne diseases of livestock in the Middle East and North Africa: a review. Insects 19:83–93. 10.3390/insects12010083 10.3390/insects12010083 PMC7835866 33477991

[CR44] Ravindran R, Hembram PK, Kumar GS, Kumar KG, Deepa CK, Varghese A (2023) Transovarial transmission of pathogenic protozoa and rickettsial organisms in ticks. Parasitol Res 122:691–704. 10.1007/s00436-023-07792-9 36797442 10.1007/s00436-023-07792-9 PMC9936132

[CR45] Renald E, Buza J, Tchuenche JM, Masanja VG (2023) The role of modeling in the epidemiology and control of lumpy skin disease: a systematic review. Bull Natl Res Cent 47:141–153. 10.1186/s42269-023-01111-z

[CR46] Rouby SR, Hussein KH, Aboelhadid SM, El-Sherif AM (2017) Role of rhipicephalus annulatus tick in transmission of lumpy skin disease virus in naturally infected cattle in Egypt. Adv Anim Vet Sci 5:185–191. 10.17582/journal.aavs/2017/5.4.185.191

[CR47] Saleh AG, Badr Y, Abas OM, Aamer WN, Inoshima Y, Rahman MM, Mokhlis HA, Abdullaziz IA (2023) Clinical, hematological, and biochemical alterations associated with early and late infection of lumpy skin disease in cattle in Egypt. Alex J Vet Sci 76:131–140. 10.5455/ajvs.104181

[CR48] Sanz-Bernardo B, Haga IR, Wijesiriwardana N, Basu S, Larner W, Diaz AV, Langlands Z, Denison E, Stoner J, White M, Sanders C (2021) Quantifying and modeling the acquisition and retention of lumpy skin disease virus by hematophagus insects reveals clinically but not subclinically affected cattle are promoters of viral transmission and key targets for control of disease outbreaks. J Virol 95:10–128. 10.1128/jvi.02239-20 10.1128/JVI.02239-20 PMC8104101 33568514

[CR49] Sanz-Bernardo B, Suckoo R, Haga IR, Wijesiriwardana N, Harvey A, Basu S, Larner W, Rooney S, Sy V, Langlands Z, Denison E (2022) The acquisition and retention of lumpy skin disease virus by blood-feeding insects is influenced by the source of virus, the insect body part, and the time since feeding. J Virol 96:e00751-e822. 10.1128/jvi.00751-22 35867566 10.1128/jvi.00751-22 PMC9364806

[CR50] Sariya L, Paungpin W, Chaiwattanarungruengpaisan S, Thongdee M, Nakthong C, Jitwongwai A, Taksinoros S, Sutummaporn K, Boonmasawai S, Kornmatitsuk B (2022) Molecular detection and characterization of lumpy skin disease viruses from outbreaks in Thailand in 2021. Transbound Emerg Dis 69(1–15):e2145–e2152. 10.1111/tbed.14552 35396931 10.1111/tbed.14552

[CR51] Selim A, Manaa E, Khater H (2021) Seroprevalence and risk factors for lumpy skin disease in cattle in Northern Egypt. Trop Anim Health Prod 53:350–358. 10.1007/s11250-021-02786-0 34105025 10.1007/s11250-021-02786-0

[CR52] Sharawi SS, Abd El-Rahim IH (2011) The utility of polymerase chain reaction for diagnosis of lumpy skin disease in cattle and water buffaloes in Egypt. Rev Sci Tech 30:821–830. 10.20506/rst.30.3.2075 10.20506/rst.30.3.2075 22435194

[CR53] Sprygin A, Pestova Y, Wallace DB, Tuppurainen E, Kononov AV (2019) Transmission of lumpy skin disease virus: a short review. Virus Re 269(1–8):197637. 10.1016/j.virusres.2019.05.015 10.1016/j.virusres.2019.05.015 31152757

[CR54] Sudhakar SB, Mishra N, Kalaiyarasu S, Jhade SK, Singh VP (2022) Genetic and phylogenetic analysis of lumpy skin disease viruses (LSDV) isolated from the first and subsequent field outbreaks in India during 2019 reveals close proximity with unique signatures of historical Kenyan NI-2490/Kenya/KSGP-like field strains. Transbound Emerg Dis 69:51–62. 10.1111/tbed.14322 10.1111/tbed.14322 34529889

[CR55] Tuppurainen ES, Lubinga JC, Stoltsz WH, Troskie M, Carpenter ST, Coetzer JA, Venter EH, Oura CA (2013) Mechanical transmission of lumpy skin disease virus by Rhipicephalus appendiculatus male ticks. Epidemiol Infect 141:425–430. 10.1017/S0950268812000805 22717050 10.1017/S0950268812000805 PMC9152053

[CR56] Tuppurainen ES, Venter EH, Coetzer JA, Bell-Sakyi L (2015) Lumpy skin disease: attempted propagation in tick cell lines and presence of viral DNA in field ticks collected from naturally-infected cattle. Ticks Tick-Borne Dis 6:134–140 25468765 10.1016/j.ttbdis.2014.11.002 PMC4329317

[CR57] Tuppurainen ES, Venter EH, Shisler JL, Gari G, Mekonnen GA, Juleff N, Lyons NA, De Clercq K, Upton C, Bowden TR, Babiuk S (2017) Capripoxvirus diseases: current status and opportunities for control. Transbound Emerg Dis 64:729–745. 10.1111/tbed.12444 26564428 10.1111/tbed.12444 PMC5434826

[CR58] Vidanović D, Šekler M, Petrović T, Debeljak Z, Vasković N, Matović K, Hoffmann B (2016) Real-time PCR assays for the specific detection of field Balkan strains of lumpy skin disease virus. Acta Vet 66:444–454

[CR59] Walker AR, Bouattour A, Camicas J-L, Estrada-Peña A, Horak I, Latif AA, Pegram RG, Preston PM (2003) Ticks of domestic animals in Africa: a guide to identification of species. Bioscience Reports, Edinburgh, United Kingdom, pp 1–221

[CR60] Wang H, Kong Y, Mei L, Lv J, Wu S, Lin X, Han X (2021) Multiplex real-time PCR method for simultaneous detection and differentiation of goat pox virus, sheeppox virus, and lumpy skin disease virus. J AOAC Int 104:1389–1393. 10.1093/jaoacint/qsab040 33769495 10.1093/jaoacint/qsab040

[CR61] World Organization for Animal Health (WOAH) (2023) Lumpy skin disease. In: Manual of diagnostic tests and vaccines for terrestrial animals. https://www.oie.int/en/what-we-do/standards/codes-and-manuals/terrestrial-manual-online-access/

[CR62] Yimer L (2021) Conventional and molecular tests of lumpy skin disease. J Anim Vet Adv 20:15–31

[CR63] Zeedan GG, Hassanain MA, Shaapan RM (2014) Isolation of parapoxviruses from skin lesion of man and animals in middle Egypt. Glob Vet 12:19–25. 10.5829/idosi.gv.2014.12.01.81156

[CR64] Zeedan GS, Mahmoud AH, Abdalhamed AM, El Abd KA, Khafagi MH, Abou Zeina HA (2019) Detection of lumpy skin disease virus in cattle using real-time polymerase chain reaction and serological diagnostic assays in different governorates in Egypt in 2017. Vet World 12:1093–1100. 10.14202/vetworld.2019.1093-1100 31528038 10.14202/vetworld.2019.1093-1100 PMC6702561

[CR65] Zeedan GS, Mahmoud AH, Abdalhamed AM, Ghazy AA, El-Razik KA (2020) Rapid detection and differentiation between sheep pox and goat pox viruses by real-time qPCR and conventional PCR in sheep and goat in Egypt. World Vet J 25:80–87. 10.36380/scil.2020.wvj11

