# Supplementary figures and images for: Molecular detection of lumpy skin disease virus in naturally infected cattle and buffaloes: unveiling the role of tick vectors in disease spread
Source: Vet Res Commun. 2024 Oct 8;48(6):3921–39. doi: 10.1007/s11259-024-10541-7 (PMC11538203; doi:10.1007/s11259-024-10541-7)

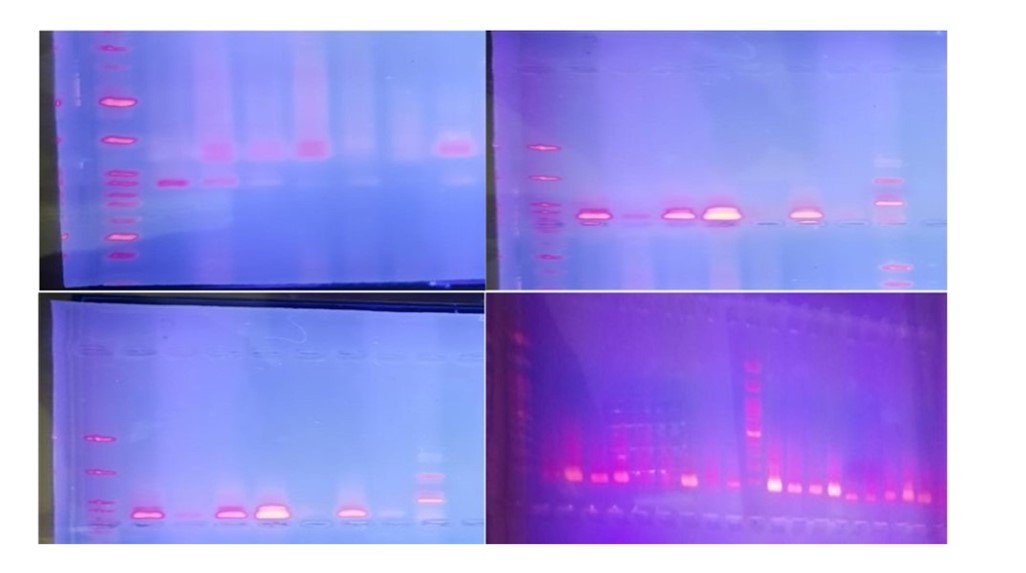

Supplement: Supplementary file 1 — Supplementary file1 (JPG 67 KB) Supplementary Figures Figs Multiplex PCRfor amplified products of LSDV isolated from skin biopsy, blood, and ticks'samples using unique primers for P32, VP32, G protein, and viral fusion proteingenes. Lane M: Marker with molecular weightmarker (1200-3000 bp), Positive at 192bp, 412 bp, and 554 bp beside NegativeControl; and Positive control for LSDV. [file 11259_2024_10541_MOESM1_ESM.jpg]
